# Effects of a Brief Stair-Climbing Intervention on Cognitive Performance and Mood States in Healthy Young Adults

**DOI:** 10.3389/fpsyg.2019.02300

**Published:** 2019-10-15

**Authors:** Andreas Stenling, Adam Moylan, Emily Fulton, Liana Machado

**Affiliations:** ^1^Department of Psychology and Brain Health Research Centre, University of Otago, Dunedin, New Zealand; ^2^Brain Research New Zealand, Auckland, New Zealand; ^3^Department of Psychology, Umeå University, Umeå, Sweden

**Keywords:** acute exercise, executive function, mood states, physical activity, stair climbing

## Abstract

**Objective:**

Previous studies focused on the benefits of acute exercise on cognition and mood have mostly used specialized laboratory-based equipment, thus little is known about how such protocols generalize to naturalistic settings. Stair climbing is a simple and readily accessible means of exercise that can be performed in naturalistic settings (e.g., at home or at the workplace). In the present study we examined the effects of stair-climbing intervals on subsequent cognitive performance and mood in healthy young adults.

**Method:**

Thirty-two undergraduate students (*M*_age_ = 19.4 years, *SD* = 1.3; 21 females) completed a controlled randomized crossover trial with session order counterbalanced across participants. Participants visited the lab on two occasions, one week apart, and completed one control session (no exercise) and one stair-climbing session (3 × 1 min stair-climbing intervals) with cognitive performance and mood assessed at the end of each session.

**Results:**

Repeated measures ANCOVA revealed that males (Hedges’ *g*_av_ = 0.45) showed better switching performance following the stair climbing but females (Hedges’ *g*_av_ < 0.03) did not. Participants felt more energetic (Hedges’ *g*_av_ = 1.05), less tense (Hedges’ *g*_av_ = 0.61), and less tired (Hedges’ *g*_av_ = 0.43) following the stair climbing. In addition, higher exercise intensity during the stair climbing predicted better subsequent switching performance and higher energetic ratings.

**Conclusion:**

These findings indicate that short bouts of stair climbing in a naturalistic setting can induce cognitive benefits for more challenging tasks, albeit only in males, indicating a sex-specific effect. Short bouts of stair climbing can be a practical approach to increase feelings of energy in daily life.

## Introduction

A growing body of research has investigated the effects of a single session of acute exercise to boost cognitive function (Lambourne and Tomporowski, 2010; Chang et al., 2012; Basso and Suzuki, 2017). Although empirical studies have shown mixed findings, meta-analyses and systematic reviews have revealed small positive effects of acute exercise on cognitive performance in samples ranging from children to older adults (Etnier et al., 1997; Sibley and Etnier, 2003; Lambourne and Tomporowski, 2010; Chang et al., 2012; McMorris and Hale, 2012; Verburgh et al., 2014; Ludyga et al., 2016). Acute exercise seems to be particularly beneficial for complex cognitive abilities, such as executive functions (Chang et al., 2012; Verburgh et al., 2014). For example, inhibitory control and switching have been shown to benefit from short bouts of acute exercise (Pesce and Audiffren, 2011; Verburgh et al., 2014; Hwang et al., 2016; Tsai et al., 2016). Although multiple studies have demonstrated positive effects of acute exercise on executive functions, the exercise has typically been carried out using specialized equipment (e.g., treadmill or cycle ergometer). Thus, research investigating the effects of acute bouts of exercise that can be carried out in naturalistic settings remains scarce.

Research also indicates that short bouts of exercise can increase positive and reduce negative mood states (Yeung, 1996; Liao et al., 2015; Basso and Suzuki, 2017). Effects have been observed immediately after and lasting up to 30 min after acute exercise sessions (Reed and Ones, 2006). However, acute exercise does not always produce mood changes in the desired direction. Exercise duration and intensity have been suggested as factors that may influence the direction and extent of the mood changes (Berger et al., 2016). In the current study, we investigated whether a short, simple, and readily accessible stair-climbing intervention could produce positive effects on executive functions and mood states in healthy young adults. We focused on executive functions and mood states as these are two of the most consistent neuropsychological effects of short bouts of exercise in laboratory settings and both are crucial for peoples’ everyday well-being and functioning (Basso and Suzuki, 2017).

Executive functions refer to a set of top–down mental processes, necessary for tasks that require concentration and attention, such as maintaining a mentally specified goal in the presence of distracting alternatives (Diamond, 2013). These functions have been associated with many important aspects of daily life, such as school and job success, mental health, and social development (Chan et al., 2008; Diamond, 2013). Thus, the effect of acute exercise on executive functions has been of particular interest in previous research (McMorris and Hale, 2012; Verburgh et al., 2014; Ludyga et al., 2016). A meta-analysis including samples of children through to older adults showed that the effect of acute exercise was larger for executive function tasks compared to tasks requiring recall or alertness/attention (McMorris and Hale, 2012). Another meta-analytic review involving a mixture of children, adolescents, and young adults found a moderate positive effect of acute exercise on executive function (Verburgh et al., 2014). In a meta-analysis focused only on moderate intensity acute exercise, small positive effects on executive function were found across samples of children through to older adults (Ludyga et al., 2016). There is also evidence that executive functions show some of the largest benefits from acute exercise when a post-exercise delay precedes cognitive testing (Chang et al., 2012). Overall, executive functions appear to be responsive to acute bouts of exercise, providing a potential intervention target.

Although the exact mechanisms underlying the cognitive improvement seen in relation to acute exercise are not fully understood, physiological changes (e.g., increases of cerebral blood flow or neuroendocrinological changes) occurring as a result of exercise are thought to play a key role (Etnier and Labban, 2012; Ando, 2016; Basso and Suzuki, 2017). A promising approach to attain the exercise intensity and physiological changes needed for cognitive benefits is high-intensity interval training (HIIT), which can be an accessible and time-efficient form of exercise. HIIT involves repeated short intervals of exercise typically at relatively high intensities (Buchheit and Laursen, 2013). There has been a growing interest regarding the possibility of HIIT as a time-efficient method to improve cognitive functions (Alves et al., 2014; Tsukamoto et al., 2016; Kao et al., 2017; Kujach et al., 2018). Different protocols have been used in previous studies ranging from ten 1-min bouts of cycling (Alves et al., 2014), four 4-min bouts of cycling (Tsukamoto et al., 2016), three 1.5-min bouts of treadmill running (Kao et al., 2017), eight 30-s intervals of cycling (Kujach et al., 2018), and ten 20-s intervals of cycling (Slusher et al., 2018). In general these studies found that the HIIT protocols had beneficial effects on both inhibitory control (Tsukamoto et al., 2016; Kao et al., 2017) and switching performance (Slusher et al., 2018), which is in line with previous findings using longer durations of exercise (Chang et al., 2012; Hwang et al., 2016). These results suggest that a long exercise session may not be necessary to achieve beneficial effects on executive functions.

Another potential benefit of acute exercise is positive mood changes. Findings from meta-analyses and systematic reviews suggest that both clinical and non-clinical samples can benefit from a single bout of exercise (Yeung, 1996; Reed and Ones, 2006; Loy et al., 2013; Basso and Suzuki, 2017). Whereas low and moderate intensity exercise has generally been associated with positive mood changes (Reed and Ones, 2006; Hogan et al., 2013; Loy et al., 2013; Legrand et al., 2018), findings are mixed related to high-intensity exercise (Ekkekakis et al., 2013). Exercise duration has also been suggested to influence the direction and extent of the effects of acute exercise on mood changes (Berger et al., 2016). Although some studies suggest that exercise duration of 7–60 min can improve mood states (Reed and Ones, 2006), most previous studies investigated the effects of 20- to 40-min exercise sessions on mood changes (Loy et al., 2013; Berger et al., 2016). Less is known about the effects of shorter exercise duration (e.g., <10 min; Loy et al., 2013; Berger et al., 2016). Exceptions are two recent studies in young adults showing that two to four short “all-out” sprints (10–30 s) over a 10- to 14-min period can decrease feelings of tension and fatigue and increase feelings of vigor and energy (Monroe et al., 2016; Nalcakan et al., 2018). However, more research is needed regarding the effects of shorter acute exercise sessions.

The current study follows on from previous research suggesting that acute bouts of exercise (e.g., HIIT) can have positive effects on inhibitory control, switching performance, and mood states (Monroe et al., 2016; Kao et al., 2017; Nalcakan et al., 2018; Slusher et al., 2018). The protocols used in previous studies, however, were carried out using specialized equipment (e.g., treadmill or cycle ergometer), with strict measurement and manipulations to keep participants at set quantified intensities (e.g., Alves et al., 2014; Tsukamoto et al., 2016; Kao et al., 2017). As such, there is a gap in the literature regarding whether the cognitive and mood benefits of time-efficient acute exercise protocols, such as HIIT, generalize to acute bouts of exercise in more naturalistic settings. Differences between modes of exercise and their effect on cognitive performance have been found in a meta-analysis (Lambourne and Tomporowski, 2010), which suggests a lack of generalizability across modes of exercise regarding effects on cognitive performance.

Previous research has also shown that the effects of exercise on cognition and mood may vary as a function of fitness or physical activity levels and sex (Budde et al., 2012; Chang et al., 2012; Hamer et al., 2012; Etnier et al., 2016; Legrand et al., 2016; McDowell et al., 2016; Tsai et al., 2016). For example, some studies indicate that acute bouts of exercise are most beneficial for cognition in fit or more physically active individuals (Budde et al., 2012; Tsai et al., 2016), whereas others have not found differential effects as a function of fitness levels (Magnié et al., 2000; Themanson and Hillman, 2006). When sex differences are found they tend to favor females over males (Colcombe and Kramer, 2003), however, some studies have shown negative effects of exercise on certain cognitive functions (implicit learning) in females (Stillman et al., 2016). Acute bouts of exercise have also been shown to increase feelings of vigor among young adult females but not males (Legrand et al., 2016). Larger overall improvements in mood responses to acute aerobic exercise among females have also been reported in previous studies (McDowell et al., 2016). Hence, physical activity levels and sex may be important variables to consider when examining relations between acute exercise, and cognition and mood; thus in the current study we included both as covariates.

In the present study we focused on short stair-climbing intervals as the mode of exercise, which provide a simple, time-efficient, and readily accessible means of exercise. Allison et al. (2017) examined the effects of brief stair-climbing protocols with the rationale that these protocols could be adapted to a typical home (or workplace). Their findings suggest that a very brief stair-climbing protocol (i.e., 3 × 1 min of vigorous ascending and descending of one flight of stairs) can produce considerable immediate physiological responses. Given that research suggests that the physiological responses to exercise need to be large to benefit cognition (Chang et al., 2012), and brief stair-climbing protocols elicit large physiological responses (Allison et al., 2017), it seems reasonable to assume that a stair-climbing protocol such as the one developed by Allison et al. (2017) could elicit improvements in cognitive performance. Furthermore, little research has been focused on the acute effects on mood states of very short exercise duration (e.g., <10 min), which we intend to investigate in the current study.

Stair-climbing intervals provide a simple mode of exercise that easily can be carried out in naturalistic settings. However, very little research has been focused on the effects of acute bouts of exercise that can be carried out in naturalistic settings. The primary purpose of the current study was to test if a very brief stair-climbing protocol (3 × 1 min intervals) could elicit immediate improvements in inhibitory control, switching, and mood states in a sample of healthy young adults. We hypothesized that inhibitory control, switching performance, and self-reported mood states would be superior following the stair-climbing protocol, compared to a control session without exercise.

A consequence of the design and aim to examine the effects in a more naturalistic setting is that we did not manipulate the exercise intensity in the present study (i.e., not beyond the verbal and visual instructions given to the participants). Hence, it was expected that the level of exercise intensity would differ between participants. A secondary purpose was therefore to assess whether exercise intensity during the stair-climbing intervals was related to cognitive performance and mood, given previous findings showing that exercise intensity can influence the duration and magnitude of the effects of exercise on cognition (Ferris et al., 2007; Kamijo et al., 2007; Chang et al., 2012; McMorris and Hale, 2012) and mood (Reed and Ones, 2006; Ekkekakis et al., 2013; Hogan et al., 2013; Loy et al., 2013; Legrand et al., 2018).

## Materials and Methods

This study was approved by the University of Otago Human Ethics Committee (reference 18/012) and all methods employed were performed in accordance with relevant guidelines and regulations. All participants gave written informed consent prior to participating.

### Participants

Inclusion criteria for participation in the current study were aged 18–24, normal or corrected-to-normal vision, no color blindness, and no neurological or psychiatric conditions (based on self-report). Initially thirty-eight undergraduate students from the University of Otago were recruited. Twenty-nine participated in association with a first or second year psychology course, and nine were recruited via online advertisement and were reimbursed NZ$40. However, data from six participants had to be excluded from the study because two participants were outside the age range of interest, three participants reported having a psychiatric disorder, and one participant did not attend their second session.

Hence, the final sample included 32 students (*M*_age_ = 19.4 years, *SD* = 1.3, range = 18–24; 21 females; 28 right handed). Prior to participation, candidate’s appropriateness to engage in physical activity was assessed using the Physical Activity Readiness Questionnaire (PAR-Q; Thomas et al., 1992). Engaging in physical activity was deemed appropriate for all candidates. On average the participants engaged in 7.9 (*SD* = 5.1) hours of physical activity (PA) per week. Mean body mass index (BMI) was 22.3 kg/m^2^ (*SD* = 2.7, range 16.1–28.6). According to international BMI guidelines (WHO, 2019), 9.4% were classified as underweight (<18.5 kg/m^2^), 81.3% were classified as normal weight (18.5–24.99 kg/m^2^), and 9.4% were classified as overweight (25–29.99 kg/m^2^).

### Procedure

In the present study we used a randomized controlled crossover design with order of session (i.e., stair climbing or control) counterbalanced across participants. Participants reported to the laboratory for two experimental sessions (stair climbing and control), at the same time of day, exactly one week apart. The heart rate (HR) monitor was placed on the participant’s left wrist to measure and record HR throughout the sessions. Initially, the participant was asked to remain seated for 5 min to obtain a measure of resting HR. Following the 5-min resting period the participant completed the stair climbing or control session.

#### Stair Climbing Session

Participants received verbal instructions regarding the ratings of perceived exertion (RPE) scale and the stair-climbing protocol, and were then shown a demonstration video of the stair-climbing phase. Participants then left the testing room with the experimenter to complete the protocol. Further instructions were provided during the stair-climbing protocol. An adapted version of the 3 × 60-s 1F stair-climbing protocol from Allison et al. (2017) was used. The protocol entailed the following phases: 2-min warm up, 45-s instructional interval, 1-min stair climbing, 1-min recovery, 1-min stair climbing, 1-min recovery, 1-min stair climbing, and 3-min cool down. For the warm up, the participant walked at a moderate pace on a flat surface from the laboratory to the stairwell and then up two flights of 12 stairs (each 17 cm in height). The participant then walked at a brisk pace back and forth along the corridor for the remainder of the 2 min. During the 45-s instructional interval, the experimenter gave final instructions for the stair-climbing and recovery phases. The three stair-climbing phases were completed on one flight of 12 stairs, measuring 17 cm in height. Participants received the following instruction adapted from Allison et al. (2017): “For the stair climbing please move vigorously. This means relatively intense but not all out, so please move up the stairs as fast as you can while taking one step at a time. Maintain control and safety at all times.” Each recovery phase involved the participant descending to the landing from their place on the stairs, and walking back and forth at a self-selected pace. RPE ratings were given immediately before (pre RPE) and after (post RPE) each stair-climbing interval. The cool down phase involved walking down two flights of stairs, then back and forth on a flat surface, all at a self-selected pace. Following cool down, the participant returned to the testing room and sat for 5 min (seated rest), after which they began the cognitive battery. Cognitive testing took approximately 7 min.

#### Control Session

All aspects of the control session were identical to the stair-climbing session, except that following the initial 5-min resting HR period, the participant remained seated in the testing room for 5 min, after which they began the cognitive battery.

### Materials and Apparatus

#### Heart Rate Monitor

Heart rate was measured and recorded every second using a Polar M430 wristwatch (Polar Electro Oy, Kempele, Finland). This device uses light emitting diodes to shine green light on the skin, with a photodiode measuring the intensity of light reflected to obtain a HR measurement^1^. Date of birth, gender, weight, and height details for each participant were entered into the device to improve measurement accuracy. Mean HR was calculated over an initial 5-min resting period prior to the stair-climbing or control protocol. Mean HR was also calculated during cognitive testing in both stair-climbing and control sessions. We calculated percentage of HRmax (%HRmax = HR/HRmax^∗^100, where HRmax = 220 – age) and percentage of HR reserve (%HRR = HR – HRmin)/HRR^∗^100, where HRmin = mean resting HR and HRR = HRmax – HRmin) as indicators of exercise intensity during the stair-climbing intervals. The %HRR represents the amount HR has increased from resting HR and is often used to determine the intensity of a particular exercise session (Swain, 2000; Pesce and Audiffren, 2011; ACSM, 2018).

#### Perceived Exertion

Ratings on the Borg RPE scale (Borg, 1998) were used as a subjective measure of intensity during the stair-climbing protocol. The Borg RPE scale has ratings from 6 (*No exertion at all*) to 20 (*Maximal exertion*), and every uneven number is anchored (e.g., 13 = *Somewhat hard*). The Borg RPE scale has been validated as a measure of exercise intensity, with multiple studies demonstrating high correlations (*r* = 0.80 − 0.90) between RPE and HR (Borg, 1998).

#### Cognitive Battery

Cognitive tests were performed using a computer-based cognitive battery, run with a 64-bit Ubuntu 16.04 operating system. Stimuli were presented on a 600 × 340 mm screen using MATLAB R2016b (The MathWorks, Natrick, MA, United States) and The Psychophysics Toolbox-3 (Brainard, 1997; Pelli, 1997). Each participant was seated with their head held in constant position using a chin rest, such that the center of the monitor was at eye level, 57 cm from the participant’s eyes. The battery included three cognitive tasks (Pro, Anti, Pro/Anti; in that order) designed to assess visuomotor processing performance (Pro), inhibitory control (Anti), and switching abilities (Pro/Anti) (Guiney et al., 2015). For all tasks, participants received both verbal and written instructions. Responses to the cognitive tasks were made using a centrally positioned button box, which had two buttons of 2 cm diameter positioned 2 cm apart (DirectIN Rotary Controller with Buttons, Empirisoft Corporation, NYC). Participants were instructed to place their left and right index fingers on the left and right buttons, respectively. Each trial began with the presentation of a white fixation dot, subtending 0.3° of visual angle on a black background in the center of the monitor. After a variable interval (400, 600, 800, 1000, or 1200 ms) a 2° green or red square (depending on the task: always green for Pro, always red for Anti, and randomly intermixed for Pro/Anti) appeared 8° to the left or right side of the fixation dot (measured to the center of the square). Participants were instructed to press the button corresponding to the same side of green squares and the opposite side of red squares. Participants were asked to respond as quickly as possible while maintaining accuracy. A 900 Hz error tone sounded for 300 ms if the participant made an incorrect response, made no response within 1500 ms, or responded in less than 100 ms (anticipation error). An interval of 500 ms occurred between trials. Variable intervals and stimulus positions were presented randomly with the constraint that each combination was equally likely to occur. In the Pro and Anti tasks, participants completed four practice trials followed by 40 test trials. In the Pro/Anti task, participants completed six practice trials and 40 test trials. Reaction time (RT) and accuracy were recorded for each trial.

#### Visual Analog Mood Scales (VAMS)

Six internal mood states (sad, energetic, tense, happy, tired, and calm; in that order) were assessed using VAMS (Machado et al., 2018). For each mood scale, the following instruction was displayed: “Click the position of the line that best represents how you feel right now.” A black 100 mm horizontal line representing (but not displaying) distinct positions from 0 to 100 was presented below the instructions. The mood state descriptor was prefixed by “Not at all” (e.g., Not at all happy) at the left end of the line, and “Extremely” (e.g., Extremely happy) at the right end. Participants responded by clicking on the horizontal line using the mouse, after which a small black vertical line appeared to display their response.

#### Physical Activity

Physical activity was assessed using the New Zealand Physical Activity Questionnaire-Short Form (NZPAQ-SF), a brief self-report questionnaire designed to assess three dimensions of physical activity behavior; frequency, duration, and intensity (McLean and Tobias, 2004). Participants were asked to provide information about physical activity in the 7 days prior to the day of reporting. Answers on the NZPAQ-SF provide estimates of duration (time/week) of brisk walking, moderate physical activity, and vigorous physical activity. Hours of physical activity per week (sum of vigorous, moderate, and walking hours per week) reported by the participant was used in the analyses. A moderate correlation (*r* = 0.41) between physical activity self-reported on the NZPAQ-SF and physical activity captured by objective HR monitoring has been reported in 18–39 year olds (Moy et al., 2008).

### Statistical Analysis

Repeated measures ANOVAs tested for a main effect of stair-climbing interval on mean and peak HR, mean %HRR, mean %HRmax, pre and post RPE, and stairs climbed. When data did not meet the assumption of sphericity, the Greenhouse–Geisser correction was used. Planned follow-up comparisons between each interval were performed using Bonferroni corrected paired samples *t*-tests.

For the cognitive tasks (Pro, Anti, and Pro/Anti), median RTs for correct responses were the main dependent variable of interest as ceiling effects on accuracy were expected (White et al., 2018). Median RTs were used to minimize the potential effects of outliers. To separate inhibitory control performance specific to the Anti task from visuomotor performance, an inhibition cost score was calculated for each participant by subtracting Pro RTs from Anti RTs (Guiney et al., 2015; White et al., 2018). To separate the switching component of the Pro/Anti task from visuomotor and inhibitory components, a switching cost measure was calculated for each participant by subtracting Anti RTs from Pro/Anti RTs (Cameron et al., 2015; White et al., 2018).

To investigate the primary purpose of the study (i.e., the effect of stair climbing on subsequent inhibitory control, switching, and mood states), repeated measures ANOVAs tested differences between the stair-climbing and control sessions in Pro, Anti, and Pro/Anti RTs, inhibition and switching cost, and the six mood state ratings. A power calculation using G^∗^Power version 3.1.9.2 (Faul et al., 2007) indicated that with a sample size of 32 and power of 0.80, the current study could detect repeated measures effects of *f* = 0.20 (corresponding to a Cohen’s *d* of 0.40) or higher assuming a 0.70 correlation between the repeated measures (White et al., 2018). Subsequently, we included physical activity and sex as covariates in repeated measures ANCOVAs. Physical activity was calculated as an average of the hours per week of physical activity reported at each session and the continuous physical activity variable was grand-mean centered prior to the being entered into the repeated measures ANCOVAs (Schneider et al., 2015). Effect sizes are reported as partial eta squared ($\eta_{\text{p}}^{2}$) in the repeated measures ANOVAs/ANCOVAs and Hedges’ *g*_av_ for pairwise comparisons (Lakens, 2013). Effect size magnitudes of 0.01, 0.06, and 0.14 for $\eta_{\text{p}}^{2}$, and 0.20, 0.50, and 0.80 for Hedges’ *g*_av_, were used to indicate small, medium, and large effect sizes (Cohen, 1988). We also estimated repeated measures ANOVAs with sex as a between-subject factor as a sensitivity analysis. Tables 3, 4 thus presents the results from all three analyses (a) repeated measures ANOVAs, (b) repeated measures ANOVAs with sex as a between-subject factor, and (c) repeated measures ANCOVAs with physical activity and sex as covariates.

To investigate the secondary purpose of the study (i.e., the effect of exercise intensity on subsequent inhibitory control, switching, and mood states), we performed linear regression analyses using the difference (Δ) between the stair-climbing and control session as the dependent variable, %HRR as the main predictor variable, and physical activity and sex as covariates. The significance level (α) was set to 0.05 and all of the analyses were performed using JASP version 0.9.1.0 (JASP-Team, 2018).

## Results

### Stair Climbing Intensity

**FIGURE 1:**
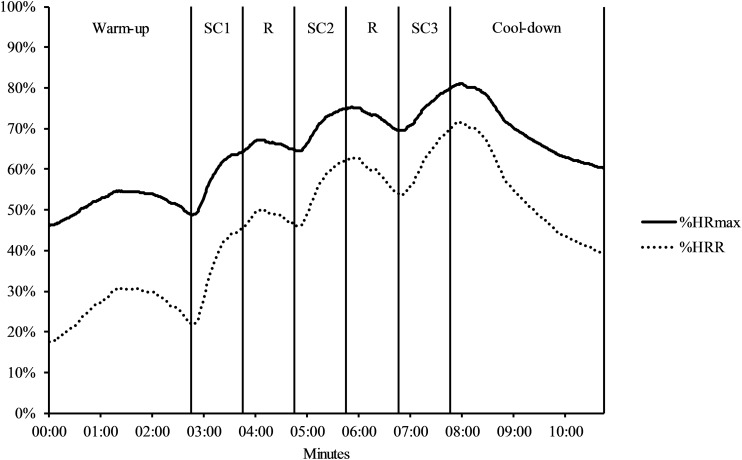
Mean %HRmax and %HRR during the stair-climbing protocol. SC, stair-climbing interval; R, recovery; HRmax, heart rate maximum; HRR, heart rate reserve.

Figure 1 graphs %HRmax and %HRR indicating a successive increase in exercise intensity across the three stair-climbing intervals. Table 1 displays descriptive statistics for the HR measures, RPE, and stairs climbed for each stair-climbing interval, and overall across the three intervals. Repeated measures ANOVAs indicated a statistically significant effect of interval on mean HR, *F*(1.38, 42.79) = 97.94, *p* < 0.001, $\eta_{\text{p}}^{2}$ = 0.76, peak HR, *F*(1.47, 45.42) = 52.12, *p* < 0.001, $\eta_{\text{p}}^{2}$ = 0.63, %HRR, *F*(1.36, 42.18) = 96.11, *p* < 0.001, $\eta_{\text{p}}^{2}$ = 0.76, %HRmax, *F*(1.38, 42.90) = 98.31, *p* < 0.001, $\eta_{\text{p}}^{2}$ = 0.76, pre RPE, *F*(2,62) = 141.10, *p* < 0.001, $\eta_{\text{p}}^{2}$ = 0.82, and post RPE, *F*(2,62) = 50.14, *p* < 0.001, $\eta_{\text{p}}^{2}$ = 0.62. Planned follow-up comparisons between each stair-climbing interval indicated a statistically significant increase with each successive interval for mean and peak HR, %HRmax, %HRR, and post RPE (*p* < 0.001 in all cases). Ratings for pre RPE showed a statistically significant increase from the first to second interval (*p* < 0.001), but not from the second to third interval (*p* = 0.532). There was no statistically significant effect of interval on the number of stairs climbed, *F*(2,62) = 2.71, *p* = 0.074, $\eta_{\text{p}}^{2}$ = 0.08. The difference in mean HR during the 5-min resting period (*M* = 78.61 vs. *M* = 79.15) was not statistically significant between sessions, *t*(31) = 0.31, *p* = 0.761, Hedges’ *g*_av_ = 0.03. However, there was a statistically significant difference in mean HR between sessions during the cognitive testing, *t*(31) = 9.29, *p* < 0.001, Hedges’ *g*_av_ = 1.13, reflecting higher mean HR during cognitive testing in the stair-climbing session (*M* = 94.46) compared to the control session (*M* = 77.81).

**TABLE 1:** Exercise intensity measures.

	**Stair climb 1**		**Stair climb 2**		**Stair climb 3**		**Overall**	
**Variable**	***M***	***SD***	***M***	***SD***	***M***	***SD***	***M***	***SD***
**Heart Rate (bpm)**								
Mean	116.5	17.3	140.8	23.3	148.8	22.7	135.3	19.7
Peak	131.9	21.6	153.6	25.0	161.7	24.1	149.1	21.4
Mean %HRmax	58.1	8.5	70.2	11.5	74.2	11.2	67.5	9.7
Mean %HRR	30.9	10.6	51.4	16.3	58.1	15.9	46.8	12.8
**RPE**								
Pre	6.7	1.0	10.6	1.6	10.9	1.9	9.4	1.3
Post	12.4	1.9	13.5	2.0	14.3	2.2	13.4	1.9
Stairs Climbed	93.1	9.8	90.6	9.8	92.6	9.7	92.1	9.0

*Values reflect the average during each stair-climbing interval; recovery phases were not included. bpm, beats per minute; HRmax, heart rate maximum; HRR, heart rate reserve; RPE, ratings of perceived exertion.*

### Effect of Stair Climbing on Cognitive Performance and Mood States

**FIGURE 2:**
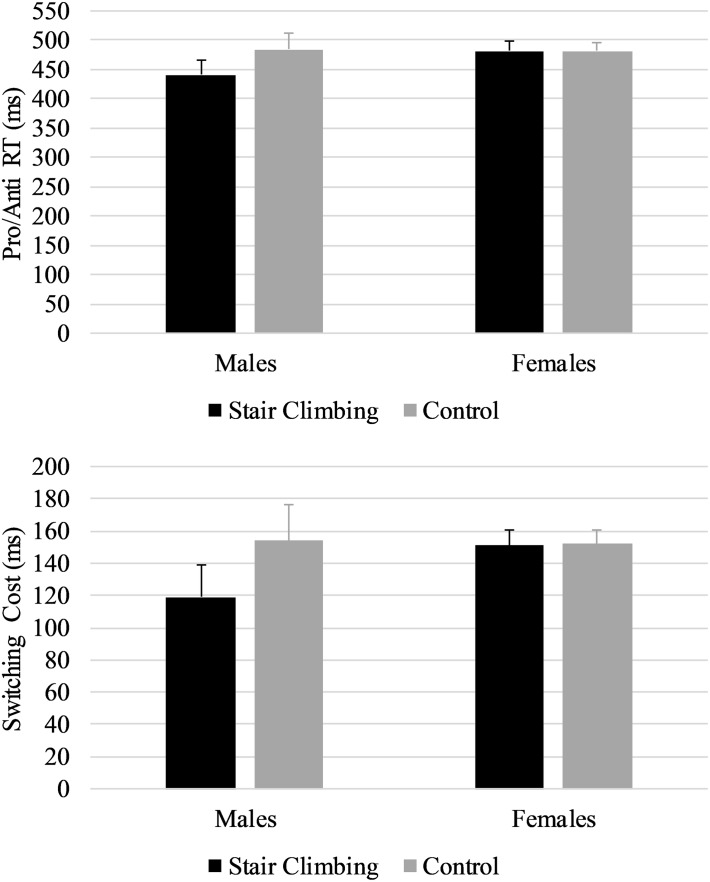
Interaction effect between sex and session for Pro/Anti RTs **(top)** and switching cost **(bottom)**. Error bars are standard errors.

Table 2 shows descriptive statistics for Pro, Anti, and Pro/Anti median RTs and accuracy, and inhibition and switching costs. As expected, accuracy was high, ranging from 95.70% to 99.84%. As summarized in Table 3, repeated measures ANOVAs (without covariates) indicated no statistically significant differences between sessions for any of the cognitive variables. However, when adding the covariates to the analyses (i.e., repeated measures ANCOVAs), a statistically significant interaction effect between session and sex was found for Pro/Anti RT and switching cost. The interaction effect indicated that males performed better on the switching task in the stair-climbing session compared to the control session (*p*s < 0.01, Hedges’ *g*_av_ = 0.45 for both Pro/Anti RT and switching cost), whereas females did not (*p*s > 0.500, Hedges’ *g*_av_ < 0.03). Figure 2 depicts these interaction effects. Session order was not counterbalanced with respect to sex; therefore, we performed a sensitivity analysis by removing the last female and male participant to enter into the study, such that within each sex group an equal number of participants commenced with the control session and stair-climbing session, and ran the analyses again. The results were consistent with the results obtained with the full sample. The results from the repeated measures ANOVAs with sex as a between-subject factor mirrored the results from the repeated measures ANCOVAs (Table 3).

**TABLE 2:** Cognitive performance and mood states in the control and stair climbing sessions.

	**Control**		**Stair Climbing**	
	***M***	***SD***	***M***	***SD***
**Cognitive Performance**				
Pro RTs (ms)	287	27	289	23
Pro Accuracy (%)	99.77	0.74	99.84	0.88
Anti RTs (ms)	330	37	328	34
Anti Accuracy (%)	98.67	1.68	99.30	1.31
Pro/Anti RTs (ms)	483	73	468	79
Pro/Anti Accuracy (%)	95.86	4.43	95.70	4.46
Inhibition Cost	44	25	39	25
Switching Cost	152	54	140	56
**Mood States**				
Sad	14.88	19.28	12.91	17.22
Energetic	39.91	22.62	62.47	19.16
Tense	41.59	23.89	26.53	24.15
Happy	57.03	16.66	62.28	16.88
Tired	47.69	27.60	36.47	23.29
Calm	56.75	19.94	56.72	18.41

*RT, reaction time; ms, milliseconds.*

**TABLE 3:** **(a)** Repeated Measures ANOVAs (RM-ANOVA), **(b)** 2(session) × 2 (sex) RM-ANOVA, and **(c)** Repeated Measures ANCOVAs (RM-ANCOVA) on the cognitive variables.

	**Pro RT**			**Anti RT**			**Pro/Anti RT**			**Inhibition Cost**			**Switching Cost**		
	***F***	***p***	**$\eta_{\text{p}}^{2}$**	***F***	***p***	***$\eta_{\text{p}}^{2}$***	***F***	***p***	***$\eta_{\text{p}}^{2}$***	***F***	***p***	***$\eta_{\text{p}}^{2}$***	***F***	***p***	***$\eta_{\text{p}}^{2}$***
**(a) RM-ANOVA^a^**															
Session	0.43	0.515	0.01	0.21	0.649	0.01	2.63	0.115	0.08	1.51	0.229	0.05	2.53	0.122	0.08
**(b) RM-ANOVA^b^**															
Session	0.27	0.606	0.01	0.67	0.420	0.02	6.05	0.020	0.17	2.21	0.147	0.07	5.07	0.032	0.15
Session^∗^Sex	0.09	0.763	0.00	1.46	0.236	0.05	6.40	0.017	0.18	1.06	0.312	0.03	4.45	0.043	0.13
**(c) RM-ANCOVA^c^**															
Session	0.08	0.781	0.00	1.06	0.312	0.04	9.05	0.005	0.24	2.12	0.156	0.07	7.58	0.010	0.21
Session * Sex	0.01	0.926	0.00	0.87	0.359	0.03	6.05	0.020	0.17	0.89	0.353	0.03	4.84	0.036	0.14
Session * Physical activity	0.25	0.620	0.01	0.23	0.638	0.01	0.15	0.701	0.01	0.00	0.991	0.00	0.49	0.491	0.02

*^*a*^*df* = 1,31, ^*b*^*df* = 1,30, ^*c*^*df* = 1,29. RT, reaction time.*

Given that previous research has found that acute bouts of exercise may be more beneficial for fit and more physically active individuals (Budde et al., 2012; Tsai et al., 2016), we also examined differences in physical activity levels between males and females. Males reported higher physical activity levels than females (*M*_males_ = 10.5 h/week, *SD* = 5.7 vs. *M*_females_ = 6.5 h/week, *SD* = 4.3), *t*(30) = 2.184, *p* = 0.037, Hedges’ *g*_av_ = 0.79. To examine whether sex-specific differences in physical activity levels might explain the observed differences in switching performance, we conducted additional analyses on a sub-sample of participants matched on their levels of physical activity (while maintaining session order counterbalanced). The sub-sample consisted of males (*n* = 8) and females (*n* = 8) with similar levels of physical activity (*M*_males_ = 8.1, *SD* = 4.0; *M*_females_ = 8.0, *SD* = 3.8). Paired samples *t*-tests within each sex group indicated better switching performance in males following stair climbing, Pro/Anti RT, *t*(7) = −2.452, *p* = 0.044, Hedges’ *g*_av_ = 0.39, *M*_diff_ = −38 ms; and switching cost, *t*(7) = −2.590, *p* = 0.036, Hedges’ *g*_av_ = 0.37, *M*_diff_ = −31 ms. There were no statistically significant differences in females between the sessions, Pro/Anti: *t*(7) = 0.128, *p* = 0.902, Hedges’ *g*_av_ = 0.03, *M*_diff_ = 2 ms; and switching cost: *t*(7) = 0.271, *p* = 0.794, Hedges’ *g*_av_ = 0.08, *M*_diff_ = 3 ms. Hence, these results mirror the results obtained with the full sample.

Descriptive statistics for VAMS in each session are provided in Table 2, and Table 4 summarizes the results from the repeated measures ANOVAs/ANCOVAs. Participants reported feeling more energetic (Hedges’ *g*_av_ = 1.05), less tense (Hedges’ *g*_av_ = 0.61), and less tired (Hedges’ *g*_av_ = 0.43) following the stair climbing, compared with the control session. There were no statistically significant interaction effects with the covariates (sex and PA) in the repeated measures ANCOVAs. The results from the repeated measures ANOVAs with sex as a between-subject factor mirrored the results from the repeated measures ANCOVAs.

**TABLE 4:** **(a)** Repeated Measures ANOVAs (RM-ANOVA), **(b)** 2(session) × 2 (sex) RM-ANOVA, and **(c)** Repeated Measures ANCOVAs (RM-ANCOVA) on mood states.

	**Sad**			**Energetic**			**Tense**			**Happy**			**Tired**			**Calm**		
	***F***	***p***	***$\eta_{\text{p}}^{2}$***	***F***	***p***	***$\eta_{\text{p}}^{2}$***	***F***	***p***	***$\eta_{\text{p}}^{2}$***	***F***	***p***	***$\eta_{\text{p}}^{2}$***	***F***	***p***	***$\eta_{\text{p}}^{2}$***	***F***	***p***	***$\eta_{\text{p}}^{2}$***
**(a) RM-ANOVA^a^**																		
Session	0.21	0.653	0.01	21.21	<0.001	0.41	9.40	0.004	0.23	1.89	0.179	0.06	5.353	0.027	0.15	0.00	0.994	0.00
**(b) RM-ANOVA^b^**																		
Session	0.02	0.881	0.00	16.53	<0.001	0.36	8.18	0.008	0.21	0.51	0.480	0.02	4.69	0.039	0.14	0.05	0.834	0.00
Session^∗^Sex	3.70	0.064	0.11	0.97	0.334	0.03	0.00	0.986	0.00	4.51	0.042	0.13	0.00	0.994	0.00	0.49	0.491	0.02
**(c) RM-ANCOVA^c^**																		
Session	0.86	0.363	0.03	2.37	0.135	0.08	5.647	0.024	0.16	0.47	0.498	0.02	1.86	0.184	0.06	0.46	0.502	0.02
Session * Sex	2.05	0.163	0.07	1.51	0.230	0.05	0.54	0.469	0.02	3.20	0.084	0.10	0.02	0.894	0.00	0.68	0.417	0.02
Session * Physical activity	0.90	0.351	0.03	0.74	0.397	0.03	4.11	0.052	0.12	0.18	0.675	0.01	0.12	0.734	0.00	0.25	0.622	0.01

*^*a*^*df* = 1,31, ^*b*^*df* = 1,30, ^*c*^*df* = 1,29.*

### Effect of Exercise Intensity on Cognitive Performance and Mood States

After controlling for the effects of sex and PA, exercise intensity (i.e., %HRR) was not a statistically significant predictor of ΔPro, ΔAnti, or Δinhibition cost. However, exercise intensity had statistically significant effects on both ΔPro/Anti RT (*b* = −1.39, *SE* = 0.67, *p* = 0.047) and Δswitching cost (*b* = −1.64, *SE* = 0.57, *p* = 0.008). The models explained 29% and 34% of the variance in ΔPro/Anti RT and Δswitching cost, respectively. Note that the dependent variable is a difference score (Δ) calculated by subtracting control session RTs from stair-climbing session RTs. Therefore, the negative regression coefficients indicate that as exercise intensity increased, the difference in RTs between the stair-climbing and control session increased (i.e., faster RTs in the stair-climbing session).

Exercise intensity was also a positive predictor of participants’ Δenergetic mood state (*b* = 0.83, *SE* = 0.39, *p* = 0.042) and the model explained 19% of the variance. The positive regression coefficient indicate that as exercise intensity increased, the difference in energetic ratings between the stair-climbing and control session increased (i.e., higher ratings in the stair-climbing session). These findings suggest that participants who executed the stair-climbing intervals with higher intensity also showed better switching performance and had higher energetic ratings. Tables 5 and 6 provide a complete description of the results from the regression analyses.

**TABLE 5:** Linear regression analysis examining the effect of stair-climbing intensity on cognitive performance.

	**ΔPro RT**			**ΔAnti RT**			**ΔPro/Anti RT**			**ΔInhibition Cost**			**ΔSwitching Cost**		
	***b***	***SE***	***p***	***b***	***SE***	***p***	***b***	***SE***	***p***	***b***	***SE***	***p***	***b***	***SE***	***p***
%HRR	0.58	0.34	0.095	0.25	0.36	0.500	–1.39	0.67	0.047	–0.33	0.32	0.307	–1.64	0.57	0.008
Sex	–1.41	9.16	0.879	8.05	9.88	0.422	52.35	18.24	0.008	9.46	8.72	0.287	44.30	15.54	0.008
Physical activity	–0.07	0.88	0.937	–0.28	0.95	0.774	–0.20	1.76	0.912	–0.21	0.84	0.809	0.09	1.50	0.958
*R*^2^	10.7%			6.9%			29.0%			7.0%			33.9%		

*Sex was coded as 0 = male, 1 = female. RT = reaction time, Δ = difference between stair-climbing and control session.*

**TABLE 6:** Linear regression analysis examining the effect of stair-climbing intensity on mood states.

	**ΔSad**			**ΔEnergetic**			**ΔTense**			**ΔHappy**			**ΔTired**			**ΔCalm**		
	***b***	***SE***	***p***	***b***	***SE***	***p***	***b***	***SE***	***p***	***b***	***SE***	***p***	***b***	***SE***	***p***	***b***	***SE***	***p***
%HRR	–0.174	0.355	0.627	0.831	0.391	0.042	0.346	0.401	0.394	0.266	0.310	0.397	–0.307	0.424	0.475	–0.272	0.384	0.485
Sex	–12.849	9.681	0.195	10.417	10.649	0.336	6.518	10.914	0.555	13.820	8.436	0.113	2.734	11.556	0.815	–7.382	10.463	0.486
Physical activity	0.733	0.932	0.438	1.439	1.026	0.172	2.278	1.051	0.039	–0.161	0.812	0.844	0.170	1.113	0.880	–0.657	1.008	0.520
*R*^2^	14.4%			18.7%			14.7%			15.8%			2.2%			4.1%		

*Sex was coded as 0 = male, 1 = female. Δ = difference between stair-climbing and control session.*

## Discussion

In the present study we examined the effects of a short, simple, and readily accessible stair-climbing intervention on subsequent cognitive performance and mood states in healthy young adults. We found that males, but not females, showed better switching performance (as evidenced by Pro/Anti RT and switching cost) following the stair climbing, compared to the control session. In addition, following the stair climbing participants reported feeling more energetic, less tense, and less tired. Moreover, higher intensity during stair climbing was associated with better switching performance and higher energetic ratings. In the sections below we discuss the implications of these findings.

Although switching performance in males benefited from the bouts of stair climbing, switching performance in females did not benefit, nor did inhibitory control in females or males. The lack of effects for inhibitory control could reflect cognitive task difficulty and exercise intensity, respectively. Regarding the latter, compared to Allison et al. (2017) and previous studies examining the cognitive benefits of HIIT protocols (Alves et al., 2014; Tsukamoto et al., 2016; Kao et al., 2017; Kujach et al., 2018; Slusher et al., 2018), the exercise intensity was on average lower among the participants in the present study. Although the participants received instructions to “move vigorously” and “as fast as you can,” not all participants reached high intensity exercise during the stair-climbing intervals. Based on recommended guidelines for exercise intensity using the %HRR (ACSM, 2018), 56% of the participants had an average %HRR above 60% (which is the lower limit for high intensity exercise based on %HRR) during one interval and 47% had two intervals with an average %HRR in the high intensity range (i.e., above 60%). The number of intervals were also fewer compared to previous studies examining cognitive benefits of HIIT protocols. It may be that the intensity needs to be higher to obtain cognitive benefits comparable to those observed in previous studies, which might be achieved by adding more stair-climbing intervals. The relative ease of the inhibitory control task in the current study compared to previous studies may also have contributed to the lack of effect of the stair-climbing protocol. The cognitive tasks used in previous studies, such as the color-word Stroop task (Alves et al., 2014; Tsukamoto et al., 2016) or the flanker task (Kao et al., 2017), may have required more cognitive resources than the Anti task used in the current study. Thus, a combination of insufficient exercise intensity and the relative ease of the inhibitory control task used in the current study may account for the lack of benefits for inhibitory control.

We found that males performed better on the switching task following the stair-climbing protocol whereas females did not. That an effect was found for the most challenging task is line with some previous findings (Pesce and Audiffren, 2011) and supports the notion that exercise may have differential effects on different cognitive functions (Etnier and Chang, 2009). However, that cognitive benefits were observed only in males was surprising given that previous studies have shown that females tend to benefit more when sex differences are observed (Etnier et al., 2016). A potential explanation for the observed differences between males and females was differences in physical activity levels (Budde et al., 2012). However, follow-up analyses of sex groups matched on physical activity levels also indicated that only males showed better switching performance following the stair climbing. These additional analyses suggest that differences in physical activity levels did not explain the observed difference between males and females in the effect of stair climbing on switching performance. Researchers have suggested that sex may be an important moderator of the effect of chronic physical activity on cognition (Colcombe and Kramer, 2003; Baker et al., 2010; Stillman et al., 2016; Barha and Liu-Ambrose, 2018). Our findings indicate that acute bouts of exercise may also have sex-specific effects on switching performance in young adults.

We also observed a positive effect of stair-climbing intensity on switching performance. This result is in line with findings from meta-analyses suggesting that higher exercise intensity is related to more cognitive benefits, particularly after a post-exercise delay period (Chang et al., 2012), which we also included in the current study. Although the mechanisms explaining why acute bouts of exercise benefit cognition remain unclear, it has been suggested that high-intensity exercise is more likely to induce the physiological changes needed for cognitive benefits, such as increases in cerebrovascular blood flow (Lucas et al., 2015), increments of brain-derived neurotrophic factor (BDNF) (Hwang et al., 2016; Jiménez-Maldonado et al., 2018), or increased activation in task-related areas in the prefrontal cortex (Kujach et al., 2018).

As hypothesized, the stair-climbing protocol produced increases in positive mood states (i.e., feeling energetic) and decreases in negative mood states (i.e., feeling tense and tired). These results are in line with the effects of HIIT protocols (Monroe et al., 2016; Nalcakan et al., 2018) and protocols with longer acute bouts of exercise (Loy et al., 2013; Chase and Hutchinson, 2015; Herring et al., 2017). That a short, simple, and readily accessible stair-climbing protocol produced similar effects as longer and more intense exercise protocols is an important finding because it shows that mood benefits of exercise can be achieved with shorter and less intense exercise in naturalistic settings. In addition, exercise intensity had a positive effect on energetic mood states, showing that the energizing effect of the stair-climbing intervals was more pronounced for those who executed the intervals with higher intensity. That very short bouts of exercise increased energy in healthy young adults is in line with previous studies in sleep deprived young women where 10 min of low-to-moderate intensity stair walking had a more energizing effect than caffeine (Randolph and O’Connor, 2017). In addition, when completed regularly the stair-climbing protocol adapted for the present study has been shown to produce long-term improvements in cardiorespiratory fitness (on average, VO2 peak increased by 7% in 6 weeks) (Allison et al., 2017). Hence, stair-climbing intervals can be worthwhile as a time-efficient method to accrue chronic health benefits, in addition to the possibility of immediate health benefits.

The present study is not without limitations. First, that we only observed cognitive benefits in males needs to be replicated in a larger sample that is balanced with regard to sex to rule out potential selection effects. Second, we did not include measures of potential mechanisms explaining the link between exercise and cognition, such as cerebrovascular blood flow. Inclusion of such measures could help elucidate when and why effects emerge. Third, we cannot rule out that day-to-day variation not associated with the intervention in for example cortisol levels or sleep may have had some impact on the results. However, such day-to-day variation are averaged out at the group level and cannot explain the differences between the stair climbing and control sessions. Fourth, the findings in the present study are restricted to healthy and relatively active young adults and cannot be generalized to other populations. Fifth, we only examined acute effects of stair climbing leaving unclear potential for long-term effects.

In light of the findings of the present study and the abovementioned limitations, there are several avenues for future research. First, an increase in the number of stair-climbing intervals might produce a higher intensity level without unduly prolonging the overall length of the protocol. Moreover, systematic manipulation of the number and duration of intervals could produce important information about potential dose-response effects. Second, including measures of potential physiological mechanisms, such as cerebrovascluar blood flow (Lucas et al., 2015), would provide a more detailed picture of the pathways linking acute bouts of exercise to cognitive and mood benefits. Third, given that performing the stair-climbing protocol regularly over six weeks improved cardiorespiratory fitness (Allison et al., 2017), it would be of interest to explore long-term effects of stair climbing on cognition and mood.

To summarize, the findings from the present study indicate that a short, simple, and readily accessible stair-climbing protocol can acutely benefit cognition and mood. Although the cognitive effects in the current study were selective in terms of sex and cognitive domain, we view the results of the present study as a promising step toward finding time-efficient exercise protocols that can be easily performed in naturalistic settings (e.g., at home or in the workplace). Such short and simple exercise protocols may prove useful in work-place interventions aimed at increasing levels of physical activity, health, and well-being (Taylor, 2005).

## Data Availability Statement

Data is available from the corresponding author upon request.

## Ethics Statement

The study was reviewed and approved by the University of Otago Human Ethics Committee (reference 18/012). The participants provided their written informed consent to participate in this study.

## Author Contributions

LM and AS designed the study. AM and EF performed the data collection. AS performed the data analyses and was responsible for drafting the manuscript. LM, AM, and EF contributed to the drafting of the manuscript. All authors approved the final version of the manuscript.

## Conflict of Interest

The authors declare that the research was conducted in the absence of any commercial or financial relationships that could be construed as a potential conflict of interest.

## Footnotes

1 https://www.polar.com/us-en/continuous_heart_rate_tracking
